# Supplementary material for: Content-rich biological network constructed by mining PubMed abstracts
Source: BMC Bioinformatics. 2004 Oct 8;5:147. doi: 10.1186/1471-2105-5-147 (PMC528731; doi:10.1186/1471-2105-5-147)
Supplement: Additional File 2 — The original results of the above study (non-essential files are deleted to keep the file size under the limit set by BMC bioinformatics). [file 1471-2105-5-147-S2.bz2 › chilibotAdditionalFile2/dip05/484ID8702798E10575/html/IL8RA_PPBP.html]

 


 **IL8RA** and **PPBP** 
  
Found 25 abstracts in PubMed, retrieved 05.  
 

 What does Google say? 
 PDF only 
| .edu only 

---

**Interactive relationship** (e.g. stimulation, inhibition, etc)

**Inhibitory relationship**- After phagocytosis of opsonized yeast, the expression of CXCR1  [ **IL8RA** ]  and CXCR2 was substantially down regulated and was accompanied by reduced Ca responses to corresponding ligands, IL 8 and neutrophil activating peptide 2 NAP 2  [ **PPBP** ] .  Ref: 12239185 Blood, 2002
**Neutral relationship**- CXCR1  [ **IL8RA** ]  and CXCR2 mediate migratory activities in response to IL 8 and other ELR CXC chemokines e.g., GCP 2 and NAP 2  [ **PPBP** ] .  Ref: 12627953 Biochemistry, 2003

**Non-interactive relationship** (e.g. studied together, co-existance, homology, etc.)

- Transendothelial migration of neutrophils in response to CXCL7  [ **PPBP** ]  or IL 8 CXCL8 was subject to modulation by CTAP III, but not CXCL4, as seen by drastic desensitization of the migratory response of neutrophils pre exposed to CTAP III, which was paralleled by selective down modulation of CXCR  [ **IL8RA** ]  2.  Ref: 12193731 J Immunol, 2002
- This was evident from experiments in which inhibition of the CTAP III processing protease and simultaneous blockade of the CXCL7  [ **PPBP** ]  high affinity receptor CXCR  [ **IL8RA** ]  2 led to complete abrogation of CTAP III mediated neutrophil adhesion.  Ref: 12193731 J Immunol, 2002
